# Establishment of Reference Norms for Lung Function Parameters of Healthy Sri Lankan Tamils

**DOI:** 10.1155/2019/2169627

**Published:** 2019-12-19

**Authors:** Mathanki Sooriyakanthan, Savithri Wimalasekera, Sivapalan Kanagasabai

**Affiliations:** ^1^Department of Physiology, Faculty of Medicine, University of Jaffna, Jaffna, Sri Lanka; ^2^Department Physiology, Faculty of Medical Sciences, University of Sri Jayewardenepura, Nugegoda, Sri Lanka

## Abstract

Spirometry and Peak Expiratory Flow Rate (PEFR) are important measurements in diagnosing and monitoring of COPD and asthma. Ethnic specific reference equations are necessary in interpretation of these parameters. However, equations for Sri Lankan Tamil adults are not available. This study aims to establish reference equations for lung function parameters of Sri Lankan Tamils. A descriptive cross sectional study was carried out in all 5 districts of Northern Sri Lanka. Participants were selected by cluster sampling. Base line data were obtained by a questionnaire. Height, sitting height, weight, arm span, mid arm circumference, and chest expansion were measured. Respiratory functions were assessed by a calibrated spirometer (Cosmed Micro Quark, Italy) and Wright compatible peak expiratory flow meter. Means, and standard deviations for Vital Capacity (VC), Forced Vital Capacity (FVC), Forced Expiratory Volume in the first second (FEV_1_), FEV_1_%, Peak Expiratory Flow Rate (PEFR) and for other forced expiratory parameters of 775 males and 687 females were determined. Lung function parameters have significant (*p* < 0.05) positive correlations with most of the anthropometric measures. Age had a significant (*p* < 0.05) negative correlation with lung function parameters in adults >20 years and positive correlation (*p* < 0.05) in 14–20 years group. Step wise multiple regression analysis was used to determine the prediction equations. Also equations based on age, height and age, arm span were derived. Age, height based equations were retested in the same population. Predicted values by the developed equations had better agreement than that of GLI 2012 equations. This can be useful in assessing the respiratory function in Sri Lankan Tamil population as there are no already existing equations.

## 1. Introduction

Prevalence of preventable chronic respiratory diseases is increasing worldwide and affects the quality of life of affected individuals [1]. Early diagnosis is important to reduce mortality due to respiratory diseases. Spirometry is the gold standard for diagnosing COPD [2]. Spirometry and Peak Expiratory Flow Rate (PEFR) are important measurements in diagnosing asthma [3].

In Sri Lanka, spirometry was not used very much in clinical practice due to inconvenience of using the manual spirometers. The introduction of flow and volume sensing turbine flow meters which are portable and generate computerized data sheets immediately upon measurements greatly facilitate the use of modern spirometers as a useful assessment tool in primary and secondary respiratory care. Ethnic specific reference equations are necessary in interpreting lung function parameters (LFP) as ethnic variations have been reported by previous studies [4–7]. Considering the Sri Lankans studies, reference norms are available for Sinhalese ethnic group [7], for children aged 8–16 years [8] and for Sri Lankan Tamil young adults aged 20–28 years [9]. Using these equations in interpretations is also not appropriate as study in Sinhalese ethnic group was done nearly two decades before with outdated spirometers. Other equations do not cover a wide age range of representative samples. Hence, Sri Lankan equations are not incorporated into modern pulmonary function machines and at present, in Sri Lanka, South Indian values are compared to find the predicted percentage of these parameters in computerized lung function reports. Global Lung Initiative (GLI) produced spirometric prediction equations that can be used in all ages globally. Data from South Asians are scarce in GLI data source [10]. Equations derived for South East Asians over predict the values for Sri Lankans children [11]. Hence, establishment of reference norms with new turbine spirometers and validating the spirometry in Sri Lanka is essential.Therefore, this study is undertaken with the aim of establishing reference norms for LFP of Sri Lankan Tamils. As Northern Province has the highest percentage of Sri Lankan Tamils than other provinces in Sri Lanka, this study was carried out in Northern Province.

## 2. Materials and Methods

A population based descriptive cross sectional study was carried out among Sri Lankan Tamils above age 13 years in Northern Sri Lanka. Ethical clearance for the study was obtained from Ethical review committee, Faculty of Medical Sciences, University of Sri Jayewardenepura, Sri Lanka. Cluster sampling method was applied to Grama Niladhari (GN) divisions: the smallest administrative division, in all 5 districts. Number of clusters in each district was determined according to the population proportion in those districts. Altogether 24 clusters were selected; and the number of clusters in each district was according to the population proportion in those districts. Calculated sample size was 1344 byusing the formula for population mean : *n* = Z^2^*γ*^2^/*d*^2^. It was decided to have participants in each age from 14–20 years as this is growing period. Thereafter, participants were grouped into age groups of half decades (21–25, 26–30, 31–35, 36–40, 41–45, 46–50, 51–55, 56–60, >60) to have participants in each group. About 50–65 participants were recruited from each cluster.

Total population in the district was divided by number of clusters to be included in that district, to find the sampling interval. The first GN division in the district was selected randomly. Number of population in the consequent GN divisions was added until sampling interval is achieved to find the next cluster. The same procedure was followed to find the required number of clusters in each district.

Officers working in the GN divisions were contacted and explained about this study. They were requested to inform the target population about this study and to invite them to a common place in their GN division in a particular day. The officers were requested to invite nonsmoking healthy participants to minimize the failures at the screening on the study day as the research team has to travel up to 250 kms to reach the study centre in out stations. Arrangements were done to have about 30 participants in a day. Study was done for 2-3 days in a cluster until the required number is achieved.

If the participantshad their both parents and grandparents of both maternal and paternal sides as Sri Lankan Tamil, they were recruited. Once the participants attend the study centre, they were explained about the study by principal investigator. If they consented, they were given an interviewer administered questionnaire to decide on inclusion or exclusion. A physical examination was done by a pre intern medical officer of the same sex. According to the outcome of questionnaire and physical examination, participants with diagnosed cardio pulmonary diseases, symptoms suggestive of respiratory disorders, smokers, alcoholics, pregnant women, those who had thoraco abdominal surgery, physical deformities and occupation related to high exposure of dust were excluded. Informed written consent was obtained from the participants. To include children below 18 years old, written consent was obtained from one of the parents/or guardians and assent was obtained from the child.

Height, weight, sitting height (SH), chest expansion (CE), and mid arm circumference (MAC) were measured as per the standard guidelines [12]. The procedure done by Mohanty et al. [13] in 2001 was followed in measuring arm span (AS). PEFR was measured with mini Wright compatible asma Plan peak flow meter which has a range of 50–800 L/min with an accuracy of 10%. PEFR and spirometric measurements were done as per ATS criteria [14]. Cosmed micro quark (Italy) electronic spirometer was used. Turbine flow meter was calibrated daily with 3 L syringe. Temperature and humidity of the environment was entered to change the volumes in BTPS. The participant was sitting erect with nose clip, with both feet on the floor, and facing away from the monitor while doing the spirometry. They were explained the procedure clearly with demonstrations. When they made errors in each attempt it was corrected by giving instructions and demonstration to correct the error. All anthropometric and lung function measurements were carried out at the same occasion in a participant. The test was done before lunch and atleast after 30 minutes of resting at the study site.

Data was analyzed in SPSS. Student's *t*-test was used to compare the significance of difference between means. Pearson's correlation coefficient (*r*) was used to get the relationship of each lung function parameter to anthropometric measurements. Step wise multiple regression analysis was done to find out the statistically significant independent variables and to devise the regression equations for lung function parameters.

To validate the equations, another 70 (38 males, 32 females) participants aged >20 years from the same population were recruited and spirometric measurements were carried out. The procedures and equipments used were same. VC, FVC and FEV_1_ of the new population were predicted by using the equations based on height and age. Also, the values were predicted as per the South East Asian reference values of GLI 2012. Correlation analysis was done to find the Pearson correlation between measured and predicted values. Bland Altman analysis was done to determine the limits of agreement between measured and predicted values.

## 3. Results

Out of 2088 participants attended for the study, 1711 of them were enrolled as nonsmoking healthy Sri Lankan Tamils. After screening, 28 of the participants withdrew from the study on their own due to difficulty in doing the spirometry. Among the participants, 221 of participants (94 males, 127 females) were excluded from the analysis due to poor spirometry reports after 8 attempts. Exclusion of spirometry reports were mainly due to poor efforts in performing forced expiratory manuevere, leak at the beginning of forced expiration, blowing errors, sudden or early termination during forced expiration and nonrepeatable attempts. Finally data of 775 males and 687 females were included in the analysis.

The age ranges of participants were 14–85 years in males and 14–76 years in females. The height and weight ranges were 136.2–186.5 cm, 28.4–126.8 kg in males and 138.0–181.0 cm, 31.6–111.6 kg in females respectively.  Table 1 summarizes the mean, SD of height, weight, SH and AS of males and females of each group.

In males, all mentioned parameters increased from 14 to 19 years of age, but remarkable higher rate (*p* ≤ 0.001) of increase was noticed between 14 and 15 years. Thereafter, changes are not significant but with age slow reduction occurs. After 20 years of age, weight increased up to 41–45 years age group and then followed by gradual reduction. SH and AS did not change significantly (*p* ≤ 0.05) from one age group to the next. In females, changes in these parameters are not significant.

As shown in Table 2, in males, Vital Capacity (VC) increased from 14 to 19 years of age with a significant increase of 580 ml (*p* ≤ 0.001), 300 ml (*p* ≤ 0.01) from 14 to 15 years and 15 to 16 years respectively. Then it decreased by 260 ml (*p* ≤ 0.05) from 19 to 20 years. It was followed by nonsignificant increase of about 70 ml in 21–25 years of age. Thereafter as the age increases VC reduced gradually. The mean Forced Vital Capacity (FVC) of 2.68 L in 14 years of age increased with age and reached 4.1 L in 19 years of age. The increases from 14 to 15 years (*p* ≤ 0.001), 15 to 16 years (*p* ≤ 0.05), 18 to 19 years (*p* ≤ 0.05) were significant. After 19 years of age, FVC decreased slowly with age. Changes in Forced Expiratory Volume in the first second (FEV_1_) were same as that of FVC. Slight increases and reductions occurred in an irregular pattern in FEV_1_% with age. Force expiratory Flow Rate at 25–75% (FEF_25–75_) generally increases from 14 to 20 years. After 20 years of age, FEF_25–75_ reduces with age. Mid expiratory flow rates at 75%, 50%, 25% (MEF_75_, MEF_50_, MEF_25_ and PEFR) increased initially and peak values were observed at 20 years. These parameters in females are summarized in Table 3.

Unlike males, the changes in VC and FVC, FEV_1_, FEF_25–75_of females from 14 to 20 years were very minimal but the maximum mean values were observed in 20 years of age. Then it decreased slowly with age. Slight changes in FEV_1_% were noticed between consequence age groups. Irregular increases and reductions were observed in MEF_75_, MEF_50_ and MEF_25_and after 41–45 years these values continuously reduced. PEFR did not show much difference from one age group to the next.

Correlation coefficient of lung function parameters with anthropometric measurements are summarized in Tables 4 and 5.

In 14–20 years population, VC, FVC and FEV_1_ showed statistically significant (*p* ≤ 0.05) positive correlations with all anthropometric characters except SHHR in females. The highest correlation was observed with height and it is nearly strong positive (0.747) with FVC of males. Correlations with SH and AS are moderately positive (*p* ≤ 0.001). In >20 years adults also VC and FVC showed higher correlation (moderately positive) with height, SH and arm span of males. While lung volumes correlate better with height, flow rates like FEF_25–75_, MEF_75_, MEF_50_, MEF_25_ and PEFR have the highest correlation with SH of 14–20 year group. In contrast to that, in >20 years of age group, flow rates correlate better with height. From 14 to 20 years, age correlated positively with lung parameters. But this was significant (*p* ≤ 0.05) only in males. After 20 years, age correlated negatively (*p* ≤ 0.05) with all parameters; but the correlations were moderate with FVC and FEV_1 _while other correlations were mild. Although these observations were noticed in both sexes, correlation co-efficient were higher in males than in females and also in 14 to 20 year population compared to >20 years population.

Regression equations derived by step wise regression analysis for VC, FVC, and FEV_1_ with highest *R*^2^ values (0.517–0.683 in males and 0.379–0.503 in females) consisted of anthropometric characters such as age, height, weight, SH, BMI, CE, SHHR, AS and MAC in different combinations. As all these parameters are not measured routinely in clinical practice, and Global Lung Initiative equations [10] are also based on age and height, equations were derived based on these parameters (Table 6). *R*^2^ values of both equations did not show much difference (0.034–0.064) except in females of 14 to 20 years (about 0.1 difference).

Mean, SD of the measured and predicted lung values of retesting population are summarized in Table 8.

Pearson correlations between measured and predicted values are shown in Table 9. Bland Altman analysis revealed 95% Confidence Interval (CI) for the differences between measured and predicted values of VC, FVC, and FEV_1_ (Table 10).

The limits of agreement for differences between measured and predicted values were smaller with the present equations than the GLI equations except FVC in males. Predicted FVC and FEV_1_ using present equations had higher correlations with measured values than the predicted values of GLI equations in both males and females. This shows that the new equation developed by this study has better agreement between measured and predicted values.

## 4. Discussion

Marked changes in lung function parameters of males from 14 to 15 years is associated with changes in anthropometric characters between these age groups. But the changes were minimal in females. This agrees with the findings of Neve et al. that the lung development in males occurred until the end of puberty while lung development in females completed at menarche [15]. As females attend puberty earlier than males and this study population did not have participants <14 years, the changes were not observable. This is also in consistent with Uduphille [7] who pointed out that males achieving pulmonary maturity 5 years later than females.

Correlations of lung function parameters with anthropometric characters and the highest correlations with height were in agreements with other findings [16, 17].

Comparison of lung function parameters with other ethnic groups adjusted for age and height are shown in Table 11.

Comparison of lung function parameters with other ethnic groups shows a wide range of variation between different ethnic groups. The above studies were done in healthy population as per standard guidelines. Different types of instruments used would have slightly contributed to the variations. Although smoking males were included in the Pakistani study and South Indian study, lung volumes were higher than the present values. South Indian study did not include older adults >40 years of age. This may partly contribute to the higher lung volumes of the population.

Caucasians had wider chest circumference than other ethnic groups and longer chests than that of Chinese and Indians [27]. This could be the reason for higher lung volume amongst Caucasians. Sri Lankans are smaller in size than Caucasians which explain a smaller lung and therefore lower lung volumes.

Genetic, nutritional, environmental and socio economical factors and physical activity play role in development and growth of lung. Variation in these factors among the above ethnic groups could explain the differences in lung volumes.

Males in the present study have closer lung volumes to Sri Lankan Sinhalese whereas females have little higher VC and FEV_1_ than that of Sinhalese. PEFR is higher in Sinhalese population than Sri Lankan Tamils. Sri Lankan Tamils and Sinhalese had almost similar mitochondrial genetic pattern [28]. Sri Lankans have mainly rice based diet. Altitude does no play a role in Sri Lanka. This explains the closer lung volumes in males of both populations. The study in Sinhalese was done using a bellow type spirometer whereas in the present study flow volume sensing turbine flow meter was used. Sample selection was convenient sampling in Sinhalese study and it was done in 1995. In addition to these factors, environmental changes in 2 decades may also have contributed to the small differences observed in females.

Multi ethnic reference equations of GLI 2012 [10], for South East Asians did not accurately predict the lung function parameters of Sri Lankan Tamils. Lack of data from South Asians for GLI equations [10] may explain these differences.

## 5. Conclusions

In conclusion, need for the ethnic specific equations for lung function parameters are reinforced and equations for Sri Lankan Tamil population were formed by this study. The study was done by including samples from all over the Northern Sri Lanka and equations derived were retested. Although retesting was done in a small sample it showed better agreement than GLI 2012 equations. Hence, new equations will useful in assessing the respiratory function in Sri Lankan Tamil population. It is anticipated that these study results will provide the data for GLI equations too.

## Figures and Tables

**Table 1:** Mean ± SD of anthropometric characters of males and females in each age group.

Age group (*n*)	Height (cm)	Weight (kg)	SH (cm)	AS (cm)
Males				
14 (77)	157.7 ± 7.0	43.8 ± 9.6	74.9 ± 4.1	163.0 ± 8.8
15 (53)	166.3 ± 7.3	54.6 ± 12.7	79.2 ± 4.2	174.1 ± 9.0
16 (68)	168.4 ± 7.2	54.1 ± 11.8	80.4 ± 3.8	176.5 ± 8.9
17 (56)	169.2 ± 6.6	53.6 ± 7.2	81.5 ± 3.4	177.0 ± 8.0
18 (57)	169.3 ± 6.7	58.0 ± 11.2	81.5 ± 4.6	177.5 ± 8.7
19 (47)	170.5 ± 7.7	63.8 ± 16.1	83.4 ± 3.5	179.5 ± 9.5
20 (48)	169.7 ± 7.4	60.6 ± 12.1	81.9 ± 4.3	179.1 ± 8.3
21–25 (38)	169.4 ± 6.9	62.7 ± 12.8	82.4 ± 3.2	178.2 ± 8.5
26–30 (37)	170.5 ± 7.4	72.8 ± 11.0	82.6 ± 3.4	179.5 ± 9.1
31–35 (49)	169.7 ± 5.3	72.8 ± 11.1	82.4 ± 3.2	177.8 ± 8.4
36–40 (56)	168.0 ± 7.0	74.1 ± 12.1	82.4 ± 3.2	177.8 ± 8.4
41–45 (48)	168.1 ± 6.1	74.1 ± 9.7	82.4 ± 3.0	177.3 ± 8.3
46–50 (45)	166.6 ± 8.3	72.8 ± 14.1	81.2 ± 3.9	174.2 ± 8.5
51–55 (42)	167.4 ± 6.2	69.8 ± 9.2	80.8 ± 3.6	178.4 ± 8.4
56–60 (33)	166.6 ± 7.2	71.4 ± 13.3	80.6 ± 3.7	177.5 ± 9.6
>60 (21)	164.4 ± 6.5	65.7 ± 9.8	79.4 ± 3.0	176.6 ± 8.3

Females				
14 (45)	156.5 ± 4.4	45.9 ± 8.2	75.4 ± 2.3	161.1 ± 6.5
15 (49)	157.6 ± 6.1	48.0 ± 6.6	75.8 ± 3.4	162.6 ± 7.0
16 (44)	157.9 ± 6.7	47.3 ± 9.6	76.2 ± 3.6	162.5 ± 7.2
17 (49)	156.4 ± 5.6	48.4 ± 7.1	75.6 ± 2.5	161.0 ± 5.3
18 (46)	157.1 ± 5.7	49.9 ± 7.1	76.1 ± 2.6	162.4 ± 5.8
19 (49)	156.2 ± 5.5	53.0 ± 9.8	76.1 ± 2.5	162.9 ± 7.0
20 (55)	158.9 ± 6.2	55.5 ± 13.4	77.4 ± 3.9	164.5 ± 7.8
21–25 (43)	157.9 ± 6.4	53.8 ± 13.3	77.2 ± 3.5	162.7 ± 8.1
26–30 (52)	158.0 ± 5.8	58.9 ± 12.2	77.0 ± 2.8	164.2 ± 8.6
31–35 (49)	154.5 ± 5.1	60.0 ± 10.5	75.7 ± 2.4	161.8 ± 6.5
36–40 (49)	156.0 ± 6.4	64.4 ± 12.0	76.0 ± 3.0	163.2 ± 7.0
41–45 (40)	156.4 ± 6.8	63.4 ± 13.8	75.7 ± 3.1	164.1 ± 8.0
46–50 (40)	155.2 ± 6.2	65.5 ± 14.1	75.2 ± 2.9	163.4 ± 7.2
51–55 (37)	155.0 ± 5.9	66.2 ± 9.8	75.1 ± 2.3	161.6 ± 7.6
56–60 (20)	154.9 ± 5.2	64.8 ± 14.5	74.5 ± 2.5	162.9 ± 5.2
>60 (20)	152.4 ± 6.0	57.9 ± 8.5	72.9 ± 2.2	159.1 ± 4.5

SH-sitting height, AS-arm span.

**Table 2:** Mean ± SD of lung function parameters of males in each age group.

Age group	VC (L)	FVC (L)	FEV_1_ (L)	FEV_1_%	FEF _25–75_ (L/S)	MEF_75_ (L/S)	MEF_50_ (L/S)	MEF_25_ (L/S)	PEFR (L/min)
14	2.61 ± 0.51	2.68 ± 0.53	2.35 ± 0.46	88.56 ± 4.15	2.85 ± 0.59	4.79 ± 0.98	3.26 ± 0.69	1.52 ± 0.36	327.01 ± 62.15
15	3.17 ± 0.57	3.28 ± 0.59	2.92 ± 0.5	87.78 ± 4.19	3.35 ± 0.91	5.85 ± 1.22	3.80 ± 1.05	1.83 ± 0.63	377.5 ± 68.63
16	3.48 ± 0.62	3.60 ± 0.6	3.18 ± 0.51	88.97 ± 5.76	3.85 ± 0.94	6.49 ± 1.25	4.26 ± 1.07	2.13 ± 0.68	419.56 ± 62.13
17	3.59 ± 0.49	3.72 ± 0.50	3.29 ± 0.41	88.77 ± 5.56	4.00 ± 0.84	6.77 ± 1.32	4.58 ± 0.96	2.13 ± 0.5	429.64 ± 64.69
18	3.64 ± 0.52	3.80 ± 0.56	3.23 ± 0.48	86.17 ± 6.17	3.69 ± 0.86	6.54 ± 1.56	4.23 ± 1.03	1.92 ± 0.49	445.6 ± 65.57
19	3.91 ± 0.66	4.10 ± 0.65	3.54 ± 0.54	87.17 ± 5.2	4.02 ± 0.85	6.84 ± 1.23	4.59 ± 1.05	2.16 ± 0.54	454.26 ± 65.89
20	3.65 ± 0.52	3.86 ± 0.58	3.45 ± 0.52	89.47 ± 5.20	4.27 ± 0.97	7.40 ± 1.29	4.82 ± 1.07	2.34 ± 0.67	469.58 ± 60.24
21–25	3.72 ± 0.58	3.88 ± 0.55	3.30 ± 0.48	85.03 ± 5.6	3.57 ± 0.89	6.57 ± 1.45	4.09 ± 1.08	1.83 ± 0.48	446.84 ± 73.92
26–30	3.69 ± 0.51	3.82 ± 0.49	3.26 ± 0.40	85.20 ± 4.07	3.61 ± 0.70	7.14 ± 1.41	4.24 ± 0.88	1.71 ± 0.37	480.81 ± 76.03
31–35	3.57 ± 0.55	3.70 ± 0.56	3.15 ± 0.48	85.09 ± 4.36	3.59 ± 0.88	7.18 ± 1.48	4.26 ± 1.14	1.66 ± 0.42	476.33 ± 60.19
36–40	3.43 ± 0.53	3.5 ± 0.54	3.02 ± 0.44	86.354 ± 4.48	3.66 ± 0.81	7.16 ± 1.47	4.39 ± 1.07	1.67 ± 0.38	467.1 ± 66.0
41–45	3.47 ± 0.55	3.54 ± 0.55	2.99 ± 0.43	84.56 ± 5.26	3.47 ± 0.94	7.09 ± 1.58	4.15 ± 1.12	1.58 ± 0.50	481.04 ± 67.32
46–50	3.19 ± 0.52	3.24 ± 0.52	2.75 ± 0.46	84.8 ± 4.59	3.2 ± 0.83	6.39 ± 1.39	3.90 ± 1.05	1.46 ± 0.42	449.33 ± 79.44
51–55	3.11 ± 0.59	3.17 ± 0.58	2.67 ± 0.50	84.22 ± 5.29	3.05 ± 0.86	6.32 ± 1.64	3.68 ± 1.23	1.38 ± 0.44	448.57 ± 78.44
56–60	3.05 ± 0.69	3.08 ± 0.68	2.56 ± 0.57	83.46 ± 5.76	2.86 ± 0.99	5.96 ± 1.76	3.52 ± 1.23	1.29 ± 0.47	438.18 ± 106.69
>60	2.62 ± 0.71	2.63 ± 0.73	2.15 ± 0.56	82.65 ± 5.95	2.30 ± 0.72	5.07 ± 1.42	2.91 ± 0.90	0.99 ± 0.38	367.14 ± 75.10

**Table 3:** Mean ± SD of lung function parameters of females in each age group.

Age group	VC (L)	FVC (L)	FEV_1_ (L)	FEV_1_%	FEF_25–75_ (L/S)	MEF_75_ (L/S)	MEF_50_ (L/S)	MEF_25_ (L/S)	PEFR (L/min)
14	2.54 ± 0.37	2.66 ± 0.31	2.39 ± 0.28	90.00 ± 4.22	3.00 ± 0.62	4.90 ± 0.74	3.47 ± 0.68	1.73 ± 0.51	310.67 ± 37.38
15	2.53 ± 0.40	2.63 ± 0.43	2.40 ± 0.35	91.60 ± 4.77	3.16 ± 0.63	4.94 ± 0.86	3.55 ± 0.74	1.80 ± 0.43	316.33 ± 46.66
16	2.49 ± 0.38	2.59 ± 0.43	2.38 ± 0.40	91.87 ± 4.29	3.17 ± 0.67	4.98 ± 0.75	3.57 ± 0.71	1.82 ± 0.51	311.14 ± 41.27
17	2.56 ± 0.30	2.65 ± 0.31	2.42 ± 0.30	91.35 ± 5.31	3.19 ± 0.75	5.09 ± 1.06	3.59 ± 0.79	1.83 ± 0.53	316.33 ± 47.06
18	2.67 ± 0.48	2.75 ± 0.50	2.48 ± 0.44	90.43 ± 5.64	3.22 ± 0.77	5.18 ± 1.04	3.57 ± 0.93	1.78 ± 0.54	331.3 ± 62.7
19	2.54 ± 0.31	2.67 ± 0.33	2.42 ± 0.32	90.8 ± 4.29	3.16 ± 0.79	5.11 ± 1.10	3.60 ± 0.89	1.74 ± 0.57	309.59 ± 42.47
20	2.67 ± 0.49	2.82 ± 0.49	2.54 ± 0.41	90.19 ± 5.36	3.23 ± 0.71	5.16 ± 1.21	3.65 ± 0.80	1.82 ± 0.49	324.91 ± 58.01
21–25	2.52 ± 0.52	2.69 ± 0.52	2.43 ± 0.41	90.65 ± 4.42	3.19 ± 0.68	5.32 ± 0.92	3.61 ± 0.75	1.83 ± 0.68	333.72 ± 42.09
26–30	2.45 ± 0.42	2.58 ± 0.41	2.25 ± 0.34	87.94 ± 4.83	2.74 ± 0.66	4.83 ± 1.05	3.20 ± 0.83	1.39 ± 0.36	322.69 ± 50.41
31–35	2.48 ± 0.37	2.60 ± 0.36	2.27 ± 0.29	87.33 ± 3.95	2.74 ± 0.51	5.10 ± 0.90	3.22 ± 0.65	1.34 ± 0.30	334.08 ± 53.26
36–40	2.39 ± 0.40	2.46 ± 0.40	2.13 ± 0.35	86.89 ± 4.92	2.63 ± 0.65	4.83 ± 1.04	3.13 ± 0.81	1.30 ± 0.38	317.76 ± 44.21
41–45	2.38 ± 0.30	2.45 ± 0.34	2.15 ± 0.30	87.66 ± 4.59	2.78 ± 0.63	5.31 ± 1.11	3.34 ± 0.78	1.36 ± 0.48	332.25 ± 55.35
46–50	2.18 ± 0.45	2.23 ± 0.45	1.93 ± 0.35	86.92 ± 4.20	2.42 ± 0.62	4.83 ± 1.37	2.95 ± 0.88	1.11 ± 0.26	311.75 ± 53.44
51–55	2.06 ± 0.43	2.13 ± 0.43	1.86 ± 0.36	87.54 ± 3.94	2.44 ± 0.67	4.50 ± 1.03	2.96 ± 0.77	1.14 ± 0.39	298.38 ± 51.93
56–60	1.87 ± 0.42	1.95 ± 0.45	1.69 ± 0.39	86.6 ± 3.79	2.13 ± 0.66	4.31 ± 1.31	2.65 ± 0.86	1.97 ± 0.30	272.5 ± 28.26
>60	1.70 ± 0.32	1.77 ± 0.33	1.51 ± 0.27	85.39 ± 5.09	1.93 ± 0.57	3.71 ± 0.92	2.39 ± 0.70	0.90 ± 0.30	263.00 ± 42.43

**Table 4:** Correlation co-efficient between lung function parameters & anthropometric characters in 14–20 years.

	Age	Height (cm)	Weight (kg)	BMI (kg/m^2^)	SH (cm)	AS (cm)	MAC (cm)	SHHR	CE (cm)
Females									
VC	0.107^∗^	0.529^∗^	0.408^∗^	0.223^∗^	0.525^∗^	0.521^∗^	0.284^∗^	0.054	0.153^∗^
FVC	0.130^∗^	0.558^∗^	0.437^∗^	0.240^∗^	0.546^∗^	0.553^∗^	0.312^∗^	0.046	0.153^∗^
FEV_1_	0.114^∗^	0.549^∗^	0.393^∗^	0.197^∗^	0.539^∗^	0.526^∗^	0.278^∗^	0.048	0.157^∗^
FEV_1_%	−0.048	−0.057	−0.161^∗^	−0.147^∗^	−0.053	−0.110^∗^	−0.126^∗^	0.000	0.009
FEF_25–75_	0.074	0.241^∗^	0.178^∗^	0.094	0.240^∗^	0.224^∗^	0.144^∗^	0.027	0.077
MEF_75_	0.095	0.222^∗^	0.245^∗^	0.170^∗^	0.236^∗^	0.246^∗^	0.240^∗^	0.046	0.109^∗^
MEF_50_	0.058	0.229^∗^	0.232^∗^	0.157^∗^	0.253^∗^	0.223^∗^	0.202^∗^	0.060	0.090
MEF_25_	0.014	0.206^∗^	0.033	−0.045	0.181^∗^	0.152^∗^	−0.003	−0.011	0.050
PEFR	0.072	0.305^∗^	0.282^∗^	0.174^∗^	0.311^∗^	0.307^∗^	0.239^∗^	0.043	0.143^∗^

Males									
VC	0.527^∗^	0.725^∗^	0.632^∗^	0.426^∗^	0.694^∗^	0.708^∗^	0.549^∗^	0.142^∗^	0.109^∗^
FVC	0.564^∗^	0.747^∗^	0.635^∗^	0.420^∗^	0.710^∗^	0.728^∗^	0.544^∗^	0.138^∗^	0.104^∗^
FEV_1_	0.560^∗^	0.692^∗^	0.573^∗^	0.369^∗^	0.689^∗^	0.675^∗^	0.487^∗^	0.180^∗^	0.096
FEV_1_%	−0.029	−0.143^∗^	−0.251^∗^	−0.236^∗^	−0.107^∗^	−0.159^∗^	−0.227^∗^	0.018	−0.052
FEF_25–75_	0.425^∗^	0.439^∗^	0.302^∗^	0.163^∗^	0.456^∗^	0.430^∗^	0.256^∗^	0.141^∗^	0.047
MEF_75_	0.511^∗^	0.510^∗^	0.414^∗^	0.267^∗^	0.534^∗^	0.502^∗^	0.389^∗^	0.171^∗^	0.050
MEF_50_	0.423^∗^	0.414^∗^	0.303^∗^	0.178^∗^	0.431^∗^	0.410^∗^	0.270^∗^	0.135^∗^	0.042
MEF_25_	0.355^∗^	0.383^∗^	0.206^∗^	0.071	0.395^∗^	0.361^∗^	0.156^∗^	0.117^∗^	0.020
PEFR	0.570^∗^	0.575^∗^	0.465^∗^	0.299^∗^	0.609^∗^	0.565^∗^	0.454^∗^	0.203^∗^	0.025

^∗^ *p* ≤ 0.05, SH-sitting height, AS-arm span, MAC-mid arm circumference, SHHR-sitting height to height ratio, CE-chest expansion.

**Table 5:** Correlation co-efficient between lung function parameters & anthropometric characters in >20 years.

	Age	Height (cm)	Weight (kg)	BMI (kg/m^2^)	SH (cm)	AS (cm)	MAC (cm)	SHHR	CE (cm)
Males									
VC	−0.464^∗^	0.582^∗^	0.254^∗^	−0.032	0.548^∗^	0.536^∗^	0.109^∗^	−0.014	0.240^∗^
FVC	−0.513^∗^	0.572^∗^	0.235^∗^	−0.048	0.545^∗^	0.516^∗^	0.107^∗^	−0.005	0.213^∗^
FEV_1_	−0.551^∗^	0.541^∗^	0.247^∗^	0.014	0.501^∗^	0.475^∗^	0.136^∗^	0.025	0.197^∗^
FEV_1_%	−0.140^∗^	−0.101	0.061	0.137^∗^	−0.117^∗^	−0.133^∗^	0.111^∗^	−0.026	−0.037
FEF_25–75_	−0.367^∗^	0.273^∗^	0.136^∗^	0.227^∗^	0.236^∗^	0.216^∗^	0.199^∗^	−0.035	0.093
MEF_75_	−0.292^∗^	0.329^∗^	0.288^∗^	0.154^∗^	0.306^∗^	0.292^∗^	0.235^∗^	−0.013	0.117^∗^
MEF_50_	−0.282^∗^	0.234^∗^	0.232^∗^	0.145^∗^	0.200^∗^	0.184^∗^	0.196^∗^	−0.033	0.085
MEF_25_	−0.437^∗^	0.265^∗^	0.167^∗^	0.054	0.234^∗^	0.207^∗^	0.137^∗^	−0.023	0.112^∗^
PEFR	−0.241^∗^	0.407^∗^	0.379^∗^	0.217^∗^	0.412^∗^	0.393^∗^	0.290^∗^	0.036	0.191^∗^

Females									
VC	−0.470^∗^	0.504^∗^	0.135^∗^	−0.066	0.443^∗^	0.476^∗^	−0.049	−0.073	0.226^∗^
FVC	−0.523^∗^	0.498^∗^	0.095	−0.106^∗^	0.445^∗^	0.467^∗^	−0.081	−0.064	0.218^∗^
FEV_1_	−0.581^∗^	0.476^∗^	0.084	−0.107^∗^	0.442^∗^	0.430^∗^	−0.091	−0.034	0.226^∗^
FEV_1_%	−0.223^∗^	−0.198	−0.048	0.016	−0.008	−0.130^∗^	−0.045	0.114^∗^	0.026
FEF_25–75_	−0.420^∗^	0.258^∗^	0.119^∗^	0.016	0.249^∗^	0.219^∗^	0.005	−0.013	0.150^∗^
MEF_75_	−0.290^∗^	0.302^∗^	0.194^∗^	0.079^∗^	0.296^∗^	0.285^∗^	0.062	−0.004	0.077
MEF_50_	−0.328^∗^	0.256^∗^	0.189^∗^	0.093^∗^	0.243^∗^	0.228^∗^	0.085	−0.018	0.107^∗^
MEF_25_	−0.469^∗^	0.197^∗^	−0.021	−0.108^∗^	0.218^∗^	0.112^∗^	−0.106^∗^	0.024	0.207^∗^
PEFR	−0.344^∗^	0.329^∗^	0.255^∗^	0.140^∗^	0.360^∗^	0.304^∗^	0.162^∗^	0.042	0.050

^∗^ *p* ≤ 0.05, SH-sitting height, AS-arm span, MAC-mid arm circumference, SHHR-sitting height to height ratio, CE-chest expansion.

**Table 6:** Reference equations lung function parameters based on age & height.

Parameter	Males			Females		
	Equation	SEE	Adj. *R*^2^	Equation	SEE	Adj. *R*^2^	
14–20 years						
VC (L)	0.051H + 0.091age − 6.565	0.451	0.579	0.036H + 3.153	0.340	0.277
FVC (L)	0.054H + 0.108age − 7.363	0.448	0.126	0.039H + 0.021age − 3.864	0.342	0.317
FEV_1_ (L)	0.041H + 0.1age − 5.486	0.417	0.559	0.034H + 2.964	0.304	0.299
PEFR (L/min)	3.847H + 15.76age − 493.558	59.007	0.455	2.577H + 87.954	49.33	0.090

>20 years						
VC (L)	0.046H + 0.018age − 3.677	0.469	0.453	0.033H + 0.015age − 2.234	0.365	0.396
FVC (L)	0.046H + 0.022age − 3.311	0.469	0.481	0.033H + 0.018age − 1.985	0.364	0.436
FEV_1_ (L)	0.036H + 0.021age − 2.192	0.398	0.485	0.026H + 0.018age − 1.186	0.306	0.469
PEFR (L/min)	4.28H + 1.023age − 220.293	71.489	0.185	2.34H + 1.252age + 1.128	47.32	0.186

^∗^H-height in cm. As height can not be measured accurately in people who have spinal disorders, another set of equations based on arm span and age also were derived (Table 7).

**Table 7:** Reference equations for lung function parameters based on age & arm span.

Parameter	Males			Females		
	Equation	SEE	Adj. *R*^2^	Equation	SEE	Adj. *R*^2^
<20 years						
VC (L)	0.040AS + 0.091age − 5.105	0.463	0.554	0.031AS + 0.009age − 2.545	0.341	0.269
FVC (L)	0.042AS + 0.109age − 5.719	0.465	0.599	0.033AS + 0.013age − 2.971	0.345	0.305
FEV_1_ (L)	0.032AS + 0.101age − 4.260	0.427	0.536	0.028AS + 0.01age − 2.266	0.309	0.275
PEFR (L/min)	3.009AS + 15.84 age − 379.033	59.68	442	2.199AS + 0.888age − 55.094	46.83	0.090

>20 years						
VC (L)	0.036AS + 0.022age − 2.16	0.468	0.456	0.028AS + 0.017age − 1.632	0.361	0.412
FVC (L)	0.035AS + 0.025age − 2.971	0.470	0.479	0.028AS + 0.019age − 1.421	0.358	0.453
FEV_1_ (L)	0.027AS + 0.023age − 0.943	0.400	0.481	0.022AS + 0.019age − 0.738	0.301	0.483
PEFR (L/min)	3.393AS + 1.327age − 90.520	71.22	0.191	1.991AS + 1.381age + 47.031	47.18	0.191

^∗^AS-arm span (cm).

**Table 8:** Mean (SD) of measured and predicted values of males and females.

	Males (*n* = 38)			Females (*n* = 32)		
	FVC (L)	FEV_1_ (L)	VC (L)	FVC (L)	FEV_1_ (L)	VC (L)
Measured values	3.05 ± 0.52	2.52 ± 0.53	2.97 ± 0.48	2.31 ± 0.5	2.02 ± 0.46	2.21 ± 0.43
Predicted values (present equations)	3.3 ± 0.50	2.10 ± 0.48		2.28 ± 0.34	1.99 ± 0.32	2.16 ± 0.43
Predicted values (GLI 2012)	2.92 ± 0.52	2.4 ± 0.53		2.62 ± 0.32	2.22 ± 0.33	

**Table 9:** Correlation co-efficient between measured and predicted values.

	Males (*n* = 38)			Females (*n* = 32)		
	VC (L)	FVC (L)	FEV_1_ (L)	VC (L)	FVC (L)	FEV_1_ (L)
Present equation	0.690^∗^	0.750^∗^	0.705^∗^	0.656^∗^	0.723^∗^	0.752^∗^
GLI 2012		0.728^∗^	0.685^∗^		0.637^∗^	0.747^∗^

*p* < 0.05.

**Table 10:** 95% CI (range) for the differences between measured and predicted values.

	Males (*n* = 38)			Females (*n* = 32)		
	FVC (L)	FEV_1_ (L)	VC (L)	FVC (L)	FEV_1_ (L)	VC (L)
Present equation	−0.973 to 0.678 (1.651)	−0.189 to 1.131 (1.32)	−0.855 to 0.680 (1.535)	−0.647 to 0.708 (1.355)	−0.576 to 0.644 (1.22)	−0.599 to 0.689 (1.288)
GLI 2012	−0.636 to 0.899 (1.535)	−0.716 to 0.966 (1.682)		−1.0681 to 0.445 (1.5131)	−0.808 to 0.419 (1.227)	

**Table 11:** Comparison of lung function parameters of adults of different ethnic group.

Ethnicity	Males (predicted for 45 years, 170 cm height)					Females (predicted for 45 years age, 160 cm height)				
	VC	FVC	FEV_1_	FEV_1_%	PEFR	VC	FVC	FEV_1_	FEV_1_%	PEFR
*Present study*	3.33	3.52	2.98		461.27	2.37	2.48	2.16		319.18
Sinhalese [7]		3.5	2.85		571.44		2.27	2.03		400.74
Calcutta [18, 19]	3.70	3.73	2.99	78.77	527.51	2.23	2.30	1.89	75.25	399.40
South Indians [20]		3.68	3.03	77.88			2.71	2.26	79.29	
North Indians [21]			3.05				3.34	2.49	77.38	
Pakistanian [22]		3.80	2.86				2.72	1.98		
Malaysian [23]		3.22	2.88				2.35	2.08		
Chinese [6]		3.95	3.22				2.86	2.35		
Brazilian [24]		4.02	3.35	82.13	478.51		3.03	2.54	84.16	359.80
Jordanian [25]		4.78	3.92				3.49	2.8		
Canadian [26]		4.63	3.67	79.25			3.42	2.76	80.29	
GLI 2012 [10]		3.98	2.57	74.00			2.32	1.92	74.00	

## Data Availability

The quantitative data support the finding of this study is currently under embargo as the research thesis is still being written. Data request after publication of this article will be considered by the corresponding author.
